# Do you hear what you see? Utilizing phonocardiography to enhance proficiency in cardiac auscultation

**DOI:** 10.1007/s40037-020-00646-5

**Published:** 2021-01-12

**Authors:** Bjorn Watsjold, Jonathan Ilgen, Sandra Monteiro, Matthew Sibbald, Zachary D. Goldberger, W. Reid Thompson, Geoff Norman

**Affiliations:** 1 Department of Emergency Medicine, University of Washington School of Medicine, Seattle, WA USA; 2 Center for Leadership & Innovation in Medical Education, University of Washington School of Medicine, Seattle, WA USA; 3 Health Research Methods, Evidence and Impact, McMaster University, Hamilton, ON Canada; 4 Division of Cardiology, Department of Medicine, McMaster University, Hamilton, ON Canada; 5 Division of Cardiovascular Medicine, Department of Medicine, University of Wisconsin School of Medicine and Public Health, Madison, WI USA; 6 Division of Cardiology, Department of Pediatrics, Johns Hopkins University School of Medicine, Baltimore, MD USA

**Keywords:** Clinical education, Computers, Simulation, New technology, Testing/Assessment

## Abstract

**Introduction:**

Cardiac auscultation skills have proven difficult to train and maintain. The authors investigated whether using phonocardiograms as visual adjuncts to audio cases improved first-year medical students’ cardiac auscultation performance.

**Methods:**

The authors randomized 135 first-year medical students using an email referral link in 2018 and 2019 to train using audio-only cases (audio group) or audio with phonocardiogram tracings (combined group). Training included 7 cases with normal and abnormal auscultation findings. The assessment included feature identification and diagnostic accuracy using 14 audio-only cases, 7 presented during training, and 7 alternate versions of the same diagnoses. The assessment—administered immediately after training and repeated 7 days later—prompted participants to identify the key features and diagnoses for 14 audio-only cases. Key feature scores and diagnostic accuracy were compared between groups using repeated measures ANOVA.

**Results:**

Mean key feature scores were statistically significantly higher in the combined group (70%, 95% CI 67–75%) compared to the audio group (61%, 95% CI 56–66%) (*F*(1,116) = 6.144, *p* = 0.015, *d*_*s*_ = 0.45). Similarly, mean diagnostic accuracy in the combined group (68%, 95% CI 62–73%) was significantly higher than the audio group, although with small effect size (59%, 95% CI 54–65%) (*F*(1,116) = 4.548, *p* = 0.035, *d*_*s*_ = 0.40). Time on task for the assessment and prior auscultation experience did not significantly impact performance on either measure.

**Discussion:**

The addition of phonocardiograms to supplement cardiac auscultation training improves diagnostic accuracy and heart sound feature identification amongst novice students compared to training with audio alone.

**Supplementary Information:**

The online version of this article (10.1007/s40037-020-00646-5) contains supplementary material, which is available to authorized users.

## Introduction

Developing proficiency in cardiac auscultation remains a time-honored skill, central to undergraduate and graduate medical education training programs. However, proficiency has proven difficult to develop and maintain, making cardiac auscultation a fertile area for investigation and curricular innovation [1–3]. Phonocardiograms are visual representations of heart sounds that have been used for diagnosis [4, 5] and have been advanced as a curricular approach to teach students the typical shape, timing, and duration of heart murmurs that correspond to specific disease processes [6, 7]. Initial investigations using phonocardiography as a curricular tool showed improvements in students’ ability to distinguish innocent from abnormal heart murmurs [8] and resident and attending physicians’ ability to recognize diastolic gallops (e.g., S3 and S4) [9] and to accurately identify underlying diagnoses [10].

These benefits may be due to improved learning of heart sounds that are both seen and heard compared to training by listening alone. Research into working memory provides evidence that multisensory processing (i.e., presenting stimuli using multiple sensory modalities simultaneously) improves learning when compared to single-modality stimuli [11]. For example, exposure to a sound with a conceptually related image improves recall of both the sound and image when compared to initial exposure to either modality alone [12–14]. Studies in multimedia learning explain these performance gains by two principles: *contiguity, *that proximity of different pieces of information are mutually reinforcing, and *signaling, *that one modality cues essential material in another modality to improve learning [15]. This would suggest that deliberate application of training models that combine different sensory inputs—such as the sound and waveforms that make up a phonocardiogram tracing—may improve learning outcomes.

Past applications of phonocardiography for teaching cardiac auscultation have shown benefit, yet no direct comparisons of phonocardiography to traditional audio-based auscultation have been published. Thus, it remains unclear whether broad application of phonocardiograms as an educational tool for novice learners yields additional learning benefits. We sought to compare the relative learning impact of adding visual phonocardiograms to traditional audio recordings for medical students with novice cardiac auscultation skills. In doing so, we aimed to evaluate whether the incorporation of multimodality learning impacted participants’ abilities to a) identify the specific features in recorded sounds and b) provide accurate diagnoses for these cases.

## Method

### Participants

We recruited preclinical students from the University of Washington School of Medicine in 2018 and 2019 immediately after their foundational cardiology curriculum that included both classroom content and physical examination skills pertaining to cardiac auscultation. We invited all first-year students via email (*n* = 252 in 2018; *n* = 271 in 2019) with a brief description of the study and a link to the study website to provide electronic consent. The email link used simple randomization to separate students into audio-only (“audio”) and audio-plus-phonocardiogram (“combined”) study groups. Enrollment was on a first-come, first-served basis until 45 students had completed the training module and initial assessment in each group. Due to funding, enrollment of students in 2019 capped when 45 total students had completed the first assessment. Students received gift cards after completing each of the assessments. The University of Washington Human Subjects Division approved the study (HSD IRB ID: STUDY00001300, dated 3/10/2017).

### Design

First-year medical students were randomized to one of two instructional conditions via the recruitment email. The audio group was trained on a series of seven cardiac auscultation cases, each representing a specific diagnosis using sounds only; the combined group was trained on the same cases, but sounds were presented in combination with phonocardiogram tracings. Following the training, all participants completed a 14-item assessment that used audio-only stimuli, presenting the seven cases seen in training (version 1) and seven cases with new, untrained sounds that presented the same findings and diagnosis (version 2). The assessment asked participants to identify features of the sounds they were hearing and the diagnoses that these sounds represented. A follow-up email prompted participants to complete a repeat assessment one week later.

### Materials and training

We developed a collection of 14 cardiac auscultation cases: 11 were drawn from recordings from the Cardiac Auscultatory Recording Database [16], and three were recorded from patients using the Eko Core stethoscope (Eko Health, Berkeley, CA, USA). We selected cases to have unique features discernable by auscultation alone (see Table S1 in Electronic Supplementary Material [ESM]) without the addition of physiological (e.g., dynamic auscultation) or pharmacologic maneuvers [17]. For each abnormal case, the patient’s diagnoses were made by a cardiologist and supported by echocardiography, and normal cases were recorded from patients with no known cardiac disease or abnormal findings. Cases included recordings at the right and left upper sternal borders, the left lower sternal border, and the cardiac apex. We processed each recording to remove sounds that might distort the diagnosis of interest (e.g., background noise, respiratory and gastrointestinal sounds) within WavePad Audio Editor (NCH Software, Canberra, Australia), and generated phonocardiograms from the processed sounds as time-series waveforms representing sound amplitude over a 10-second sample. Audio processing was performed using standard Apple (Apple Inc., Cupertino, CA, USA) earbuds, such that participants would not require high-fidelity equipment to listen to the intended sounds. Video phonocardiograms depicted a sweeping vertical line to indicate which portion of the waveform corresponded to the contemporaneous audio (see Fig. S1 in ESM). Two board-certified cardiologists, who are members of the study team (MS, ZG), reviewed the edited sounds and phonocardiogram videos to ensure that they were accurate representations of the intended diagnoses and that audio and video quality were of sufficient fidelity.

We built our cardiac auscultation training and assessment modules using REDCap (Research Electronic Data Capture, Vanderbilt University, Nashville, TN, USA), a web-based platform that allowed students to participate asynchronously using their own devices at a time and place of their choosing. The modules were accessible via computer web browser, on tablets, and on smartphones, and the recruitment email and module instructed participants to use headphones. The modules consisted of sound testing and consent, training, and assessment phases. On the welcome page, participants were required to discern a 50% volume difference in test tones at 200 Hz (within the range of heart sounds) accounting for differences in hearing, headphone quality, and testing environment. Students provided electronic consent and a valid university email address for follow-up.

**Fig. 1:**
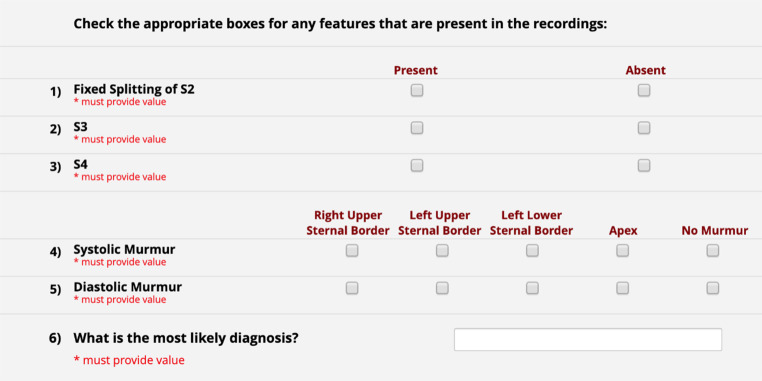
Assessment tool for features and diagnosis

Participants then completed a training module that represented the study intervention. All participants received a brief introduction to phonocardiography, demonstrated as a schematic and screenshot of abnormal phonocardiograms. Training included seven cases, each presented as a separate page, with illustrations of a patient’s chest indicating each of the four typical auscultation locations. Embedded audio links played unique location-specific sounds; participants in the combined group were provided embedded video phonocardiograms with the sounds. At the bottom of each page participants identified features and locations of the heart sounds (e.g., murmurs, gallops; see Fig. 1) using checkboxes, and then selected the most likely diagnosis from a pull-down menu. Visual feedback on the page indicated incorrect selections and confirmed correct features and diagnosis.

To gather validity evidence for response process and internal structure of our assessment instrument [18], two second-year medical students from the University of Washington who were not participants in the study pilot tested the platform. We used a think-aloud process [19] to clarify training and testing instructions, ensure training and testing materials worked on multiple web-based browsers, test whether auditory stimuli played with sufficient fidelity on multiple sets of headphones, and estimated the time spent in the training and testing. Four additional cardiology faculty, two each from the University of Washington and McMaster University, independently reviewed the 14 cases for clarity and feature identification. Case materials were amended based upon their feedback, including substituting cases and reprocessing sounds for improved clarity. After the cases and response format had been refined based on student and faculty members’ feedback, the study began enrollment in 2018.

### Measures and analysis

The first assessment phase immediately followed training and used the same format as the training cases. Fourteen cases, seven that were used in training and seven alternate versions of the same diagnoses, were presented in random order using audio-only stimuli. Students identified features using checkboxes and were required to select “present” or “absent” with no default value. We asked for diagnoses to be entered as free text in this phase to limit cueing effects [20] (see Fig. 1). After submitting responses for all 14 cases, students were provided the list of correct diagnoses alongside their answers for comparison. Participants provided demographic information including their age, sex, and whether they had worked in health care or been trained to auscultate heart sounds prior to medical school. They provided a university email address to facilitate delivery of the honorarium. One week later, participants received an email link to a second assessment containing the same 14 cases in random order, with an identical answer format. To avoid cueing, students were not told that these were cases that they had seen previously; upon completing the assessment, they were again provided with the list of correct diagnoses.

We exported de-identified data from the REDCap platform into an Excel spreadsheet (Microsoft Corporation, Redmond, WA), then analyzed using SPSS (IBM Corp. Released 2019. IBM SPSS Statistics for macOS, Version 26.0.0.1, Armonk, NY: IBM Corp.). Following the rubric outlined in Fig. 1, feature identification accuracy was calculated by correct identification of abnormal sounds (e.g., fixed split S2, S3, S4) and/or murmurs (systolic or diastolic at each of the four auscultation sites). Of 11 possible features, up to 4 abnormal findings were present in each case, with 4 of 7 cases having only a single abnormal finding. We initially calculated accuracy as correct identification of present or absent out of 11 features. To improve discrimination and reduce the ceiling effect on scores, we divided the features into three groups, extra sounds (3 features representing types of extra sounds), systolic murmurs (4 features representing location) and diastolic murmurs (also 4 features). As no case had features in more than one group, we focused scoring only on the group that was relevant to the particular case. We scored “key feature” accuracy if a participant correctly identified all features as present or absent in the relevant group for the case.

We calculated diagnostic accuracy using participants’ free-text responses. Entries frequently contained physiologic or anatomic diagnoses, such as “atrial septal defect” as the diagnosis for “fixed split S2,” or additional information, e.g., “left ventricular failure with S4.” These were adjudicated by two emergency physician members of the study team (BW, JI). We allowed misspellings and partial answers to be correct so long as the process was accurate to the intended diagnosis, and discrepancies were resolved by consensus.

We tested our hypotheses comparing the two training conditions using repeated measures ANOVA for each of the scoring formats: total numerical feature score out of 11, key feature accuracy as correct/incorrect, and diagnostic accuracy as correct/incorrect. Repeated measures ANOVA allows for multiple time points to be combined for comparison of treatment conditions, so scores were analyzed across all cases and both assessments. We used one between-subject factor of training condition (audio vs. combined) and three within-subject factors of assessment (initial vs follow-up), as well as case (seven levels, representing each unique diagnosis) and case version (trained vs untrained). We calculated Cohen’s *d*_*s*_ values for effect size [21, 22]. We then compared correlations between key feature and diagnostic accuracy by case and version. We examined demographic factors for significant effects and included prior auscultation training as a covariate. Start and end timestamps were used to estimate time on task during each assessment.

## Results

One hundred thirty-five students completed the training modules and initial assessment (*n* = 90 in 2018, *n* = 45 in 2019) and 119 participants completed the second assessment (*n* = 78 in 2018, *n* = 41 in 2019), with similar completion rates within the audio 60/68 (88%) and combined groups 59/67 (88%). Tab. 1 lists participant demographics.

**Table 1:** Demographics of first-year medical student participants

	Audio (*n* = 68)	Combined (*n* = 67)
Age	26.13 ± 3.26	24.79 ± 1.64
Sex: Male	32 (47%)	39 (58%)
Sex: Female	34 (50%)	27 (40%)
Sex: Prefer not to answer	2 (3%)	1 (1%)
Prior auscultation training	14 (21%)	9 (13%)

Group comparisons from repeated measures ANOVA are listed in Tab. 2. Our analysis excluded participants who did not complete the second assessment; of note here: these participants did not have a significantly different performance on any measure on the first assessment than participants who completed both assessments. For the remaining 119 participants, we analyzed two constructs of feature score: total score for each case (out of 11 features) and key feature score. Total scores did not differ significantly between the audio (10.14, 95% confidence interval [CI] 10.00–10.28) and combined groups (10.31, 95% CI 10.17–10.45) (*F*(1,116) = 2.830, *p* = 0.095). The combined group had higher mean key feature scores (70%, 95% CI 65–75%) than the audio group (61%, 95% CI 56–66%) and this difference of 9% (95% CI 2–16%) represented a small effect size (*F*(1,116) = 6.144, *p* = 0.015, *d*_*s*_ = 0.45). Diagnostic accuracy was also higher in the combined group (68%, 95% CI 62–73%) compared to the audio group (59%, 95% CI 54–65%) a difference of 8% (95% CI 1–16%) that also reflected a small effect size (*F*(1,116) = 4.548, *p* = 0.035, *d*_*s*_ = 0.40). On subgroup analyses, we found no significant differences between training groups in feature identification or diagnostic accuracy by assessment, case, or case version. Comparison of initial and delayed scores overall showed non-significant increases in both key feature scores (mean improvement 65.31 to 65.41%, *t* = 0.726, *p* = 0.469) and diagnostic accuracy (62.06 to 64.53%, *t* = 1.514, *p* = 0.133). The time × group interactions were not significant (key feature, *F*(1,116) = 0.174, *p* = 0.677; diagnoses, *F*(1,116) = 0.124, *p* = 0.725) suggesting that there was no difference between groups in the increases from immediate to delayed.

**Table 2:** Repeated measures ANOVA comparison of feature and diagnostic accuracy by the audio and combined training cohorts with prior auscultation training as a covariate

	Audio group (*n* = 60) ^a^		Combined group (*n* = 59)				
*Feature accuracy*							
Score ^b^	Mean	95% CI	Mean	95% CI	*F*	*p*	*D*_*s*_^e^
Total ^c^	10.14	10.00–10.28	10.31	10.17–10.45	2.830	0.095	–
Key ^d^	61%	56–66%	70%	65–75%	6.144	0.015	0.45
*Diagnostic accuracy*							
Score ^b^	Mean	CI	Mean	CI	*F*	*p*	*D*_*s*_^e^
Diagnosis	59%	54–65%	68%	62–73%	4.548	0.035	0.40

^a^ ANOVA excludes participants that did not complete both assessments; 8 participants from each group did not complete assessment 2

^b^ All scores are averaged across 14 cases per assessment and 2 assessments

^c^ Total score is out of 11 features per case

^d^ Key feature score indicates all 3 or 4 relevant features correctly identified for the case

^e^ Cohen’s *d*_*s*_ suggested cutoffs are small (0.2), medium (0.5), and large (0.8)

We examined correlations between key feature and diagnostic accuracy on a case-by-case level. For the four cases that presented normal sounds and abnormal sounds (split, gallops), the average correlation between key feature and diagnostic accuracy was 0.83 (range 0.63–0.97); all were significant at *p* < 0.001. For the three cases presenting murmurs, the average correlation was 0.36 (range 0.07–0.54), and except for the untrained version of case 5, all *p* < 0.02. Case 5 represented aortic stenosis, with systolic murmurs audible at all four positions, and had high feature accuracy (81%) with lower diagnostic accuracy (68%). Poor correlation between feature and diagnostic score was in part due to students listing mitral regurgitation as the diagnosis, which we considered incorrect given the location and character of the sounds, 21 times in the first assessment and 23 times in the second.

Finally, we looked at the time taken in learning and assessment in the two groups. Median training time was 12:40 min across all participants (interquartile range [IQR] 10:30 to 16:31), and median testing times (e.g., the time spent taking each assessment) on assessments 1 and 2 were 20:36 (IQR 15:30–32:00) and 18:19 (IQR 13:46–26:51), respectively. There were no significant differences in the average training or testing times between the two groups. Finally, we examined the correlations between diagnostic accuracy and the average total time that each group spent in both training and testing; correlations ranged from −0.10 to +0.02, and none were significant.

## Discussion

This study measured the effects of training first-year medical students’ cardiac auscultation skills using phonocardiograms, predicting that training using both auditory and visual stimuli would improve student performance compared to peers who were trained with audio alone. Students assigned to the combined phonocardiogram condition demonstrated greater accuracy for both key features and diagnosis, albeit with modest improvements for both results. Feature identification was moderately to strongly correlated with diagnostic accuracy in cases where participants were presented with normal or extra heart sounds, though the correlations between feature identification and murmurs were weak.

Our study is the first to compare the use of audio-only cases to audio with phonocardiogram videos for the purposes of learning cardiac auscultation skills. Michaels and colleagues demonstrated improved performance identifying gallops when clinicians used phonocardiograms concurrently with audio recordings of heart sounds compared to their first-pass attempt using audio alone [9]. Similarly, longitudinal curricula using digital phonocardiography to train cardiac auscultation in medical students [8], residents, and experienced clinicians [10] demonstrated interval improvement in these clinicians’ diagnostic accuracy. Importantly, these improvements were from comparisons of pre- and post-curricular assessments that included phonocardiograms, and thus the question of whether auscultation skills might have improved in the absence of these tracings had remained unanswered. Despite past evidence suggesting moderate associations between time on task with knowledge and skills outcomes related to teaching cardiac auscultation using simulation [23], our results indicate that auscultation skills might be improved using phonocardiography in a fairly time-limited intervention.

The visual format of a phonocardiogram may allow a learner to utilize working memory differently by restructuring timing and frequency information between audio and spatial information [15]. Our results are consistent with predictions that visual information presented concurrently with auditory information would improve learning.

Beyond stimuli presentation, we considered whether the assessment format influenced diagnostic accuracy. Our assessment design was similar to work by Sibbald and colleagues who demonstrated improved diagnostic performance using a checklist for heart sounds without improvement in concurrent feature identification [24]. These investigators hypothesized that this effect was driven by participants reasoning backwards from presumed diagnoses to identify features that they had marked incorrectly. Our students had low correlations between feature and diagnostic accuracy for cases where the features did not directly identify a diagnosis, such as a murmur heard at multiple locations. Our study design cannot, however, illuminate whether students used features to inform diagnoses or vice versa.

We suggest that future study might elaborate on these results by expanding the breadth and complexity of cases. This would entail both the inclusion of unedited sounds, to understand whether visual cues need to be simplified or are as effective with artifact present, and the inclusion of multiple diagnoses in each case, to reduce the ceiling effect on scores. This work would also recruit more advanced trainees and practitioners to examine the benefit relative to experience. The format of the assessment would also be subject to refinement, including randomizing students to respond first to either diagnosis or feature checklists. In this way one could investigate whether identifying features triggers a diagnostic impression or whether students search for features based on their presumed diagnosis.

### Limitations

Our study had several limitations. First, because we screened students for their ability to discriminate sample tones using computer headphones prior to enrollment, it is possible that we excluded a more generalizable group of students who had technological or hearing difficulties. This limitation applies to both study groups and should not have significantly biased our results. Second, current technology for producing phonocardiograms is very susceptible to artifact and extraneous visual noise, making interpretation of specific visual features more challenging. In an effort to limit these artifacts in our phonocardiograms, our sounds were processed to eliminate extraneous noises, which may limit the generalizability of our results to the bedside when learners use phonocardiograms in real time with real patients. Though this study was designed to investigate the potential incremental benefit of adding visualization of heart sounds to listening only, it is possible that use of the phonocardiogram alone, without audio, may have resulted in diagnostic accuracies similar to either combined stimuli or audio alone. Though we did not include this arm in the study because of sample size considerations and lack of proven educational value for our participants, this would be potentially useful to test in future investigations when heart sound visualization and filtering technology have improved, particularly since hearing limitations could be partially mitigated if this were true. Third, the change from pull-down responses to free-text diagnoses introduced extraneous information that required adjudication. Although this was intended to demonstrate generalizable performance rather than students learning the assessment, the nature of cardiac diagnosis in an online platform limits how specific a diagnosis can be. Fourth, due to the idiosyncratic ways that students interact with patients on the wards, some students may have had more exposure to cardiac auscultation than others. We would not expect this difference to have different distribution between the two groups. Finally, prior research has shown that auscultation performance—when trained using a cardiorespiratory simulator—declines when applied to the examination of real patients [25]; this was not the focus of this study, but we might expect similar effects amongst our trainees. Strengths of this investigation include the use of heart sounds recorded from actual patients, extensive review of cases by board-certified cardiologists, and the relatively large sample size of first-year medical students. Interpretations of these results are strengthened by the deliberate collection of content, response process, and internal structure validity evidence to support the scores from this novel assessment [18].

### Conclusions

The addition of phonocardiograms to supplement cardiac auscultation training improves auscultation performance amongst novice students after a fairly time-limited training intervention. Our results suggest that students would benefit from multimodality practice early in training. Refinement and exploration of these training interventions will be helpful to further enhance proficiency in cardiac auscultation and serve as a means to better understand how to optimize auditory and visual modalities in clinical training.

## Supplementary Information

Supplemental Figure 1 shows a ten-second phonocardiogram for the S3 training case. Supplemental Table 1 shows case diagnoses with representative features present at each auscultation location
