# Supplementary material for: Do you hear what you see? Utilizing phonocardiography to enhance proficiency in cardiac auscultation
Source: Perspect Med Educ. 2021 Jan 12;10(3):148–54. doi: 10.1007/s40037-020-00646-5 (PMC8187497; doi:10.1007/s40037-020-00646-5)
Supplement: Supplementary file 1 — Supplemental Figure 1 shows a ten-second phonocardiogram for the S3 training case. Supplemental Table 1 shows case diagnoses with representative features present at each auscultation location [file 40037_2020_646_MOESM1_ESM.docx]

**Table S1** Case diagnoses with representative features present at each auscultation location

| Diagnosis* | Abnormal Sound | | | Systolic Murmur Location† | | | | Diastolic Murmur Location† | | | |
| --- | --- | --- | --- | --- | --- | --- | --- | --- | --- | --- | --- |
|  | Split S2 | S3 | S4 | RUSB | LUSB | LLSB | Apex | RUSB | LUSB | LLSB | Apex |
| Normal‡ | - | - | - | - | - | - | - | - | - | - | - |
| Fixed Split S2 | + | - | - | - | - | - | - | - | - | - | - |
| S3 | - | + | - | - | - | - | - | - | - | - | - |
| S4 | - | - | + | - | - | - | - | - | - | - | - |
| Aortic Stenosis | - | - | - | + | + | + | + | - | - | - | - |
| Mitral Regurgitation | - | - | - | - | - | - | + | - | - | - | - |
| Aortic Regurgitation | - | - | - | - | - | - | - | - | + | + | - |

* Each of the seven diagnoses was represented by a single case in the training phase; a second unique case was added to the assessment, for a total of 14 cases. For each box, (+) indicates the presence and (-) the absence of the abnormal sound or murmur in the case and at the specified location.

† RUSB: Right upper sternal border, LUSB: Left upper sternal border, LLSB: Left lower sternal border.

‡ Gray boxes indicate relevant features for key feature scoring for each diagnosis group.

**Fig. S1** Ten-second phonocardiogram for S3 training case*


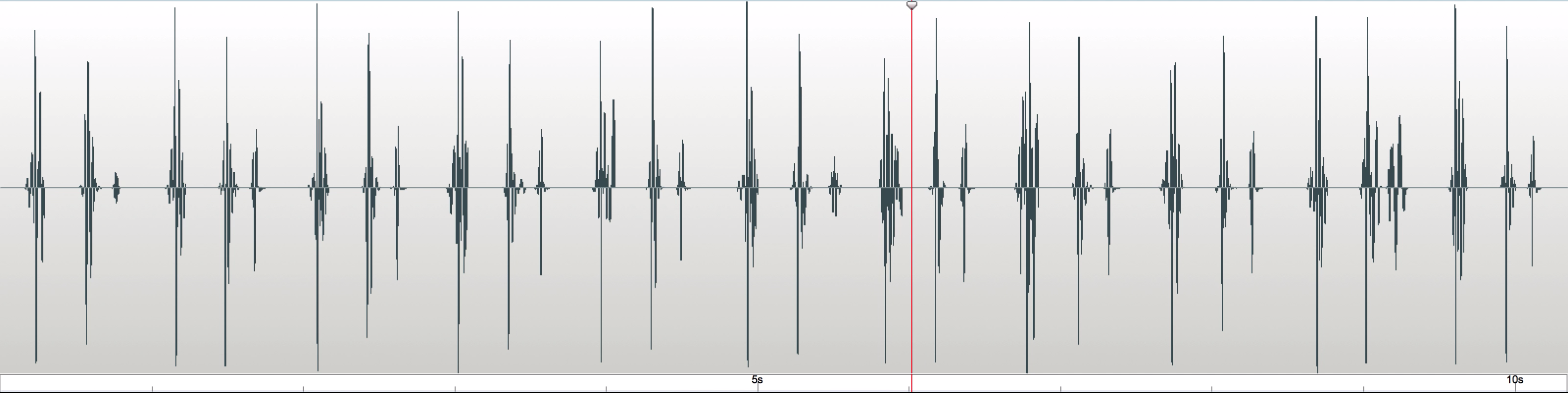


S2

S1

S3

* S1, S2, S3 labeled for beat 6. Red vertical line indicates time point at 6-second mark, during systole of beat 7.
